# Fibroblast growth factor 21 and fructose dynamics in humans

**DOI:** 10.1002/osp4.317

**Published:** 2019-02-19

Following the publication of the above article, the name of one of the authors was incorrectly presented. E. M. Flier should be shown as E. Maratos‐Flier.

The authors sincerely apologize to the readers for this error.
